# The optimum sevoflurane concentration for supraglottic airway device Blockbuster™ insertion with spontaneous breathing in obese patients: a prospective observational study

**DOI:** 10.1186/s12871-017-0449-5

**Published:** 2017-11-28

**Authors:** Haixia Wang, Xue Gao, Wei Wei, Huihui Miao, Hua Meng, Ming Tian

**Affiliations:** 1 Department of Anesthesiology, Beijing Friendship Hospital, Capital Medical University, Beijing, China; 2 Department of General Surgery, Beijing Friendship Hospital, Capital Medical University, Beijing, China

**Keywords:** Obesity, Spontaneous breathing, Sevoflurane, Supraglottic airway device

## Abstract

**Background:**

Airway management of the obese patient presenting for surgery is more likely to be a challenging problem. Supraglottic airway device has been adopted as a bridge to connect ventilation and tracheal intubation in obese patients who would be suffered with difficult intubation. The optimum sevoflurane concentration for supraglottic airway device insertion allowing spontaneous breathing in 50% of obese patients (ED_50_) is not known. The purpose of this study was to determine the ED_50_ of sevoflurane for supraglottic airway device Blockbuster™ insertion with spontaneous breathing in obese patients requiring general anesthesia.

**Methods:**

Thirty elective obese patients (body mass index 30-50 kg/m^2^) undergoing bariatric surgery were recruited in this study. The predetermined target sevoflurane concentration (initiating at 2.5% with 0.5% as a step size) was sustained for >5 min using a modified Dixon’s up-and-down method, and then the supraglottic airway device Blockbuster™ was inserted. The patient’s response to supraglottic airway device insertion was classified as either ‘movement’ or ‘no-movement’. The ED_50_ of sevoflurane were determined by calculating the midpoint concentration of crossover point from ‘movement’ or ‘no-movement’ response.

**Results:**

The ED_50_ of sevoflurane for supraglottic airway device Blockbuster™ insertion in obese patients calculated using up-and-down method were 2.50 ± 0.60%. The ED_50_ and ED_95_ (95% confidence interval) obtained by probit regression analysis were 2.35 (1.28–3.42) % and 4.03 (3.16–17.83) % for supraglottic airway device Blockbuster™ insertion, respectively.

**Conclusion:**

We conclude that the optimum end-tidal sevoflurane concentration required for the supraglottic airway device Blockbuster™ insertion allowing spontaneous breathing in 50% of obese patients (ED_50_) is 2.5 ± 0.6%.

**Trial registration:**

Chinese Clinical Trial Registry, ChiCTR-IPR-16009071, Registered on 24 August 2016.

## Background

Obesity results in higher risk of difficult mask ventilation as well as tracheal intubation and oxygen desaturation rapidly following the cessation of breathing [1–3]. The difficult airway management guideline recommends awake intubation should be gold standard in patients with anticipated difficult airways [4]. Awake intubation allows spontaneous breathing to keep with continuous oxygenation and avoid the passive situation of ‘can’t intubate, can’t ventilate’ for obese patients. However, awake intubation may be difficult to perform in obese patients and cause distress to the patients [5]. Recently, Supraglottic airway devices have advanced roles include the following: airway management in obese and higher risk patients; as a conduit for facilitating safer tracheal intubation routinely or during difficulty; airway rescue after failed intubation [6]. Adoption of the general approach with a supraglottic airway device is conclusive to improve the quality and safety of airway management in obese patients.

The supraglottic airway device Blockbuster™ (Tuo Ren Medical Instrument Co., Ltd., Changyuan City, China) is a second-generation supraglottic airway device [7]. The device is a pharyngeally inserted airway device with an anatomically shaped airway tube. It is designed to provide high airway seal pressures around the laryngeal opening and has a separate tract to insert a stomach tube to prevent gastric aspiration. This modified form of the supraglottic airway device has been specially designed to facilitate fiberoptic-guided or blind tracheal intubation.

Recently, the supraglottic airway device has been established as a viable device for ventilation, and then facilitating safer tracheal intubation in obese patients [8–11]. Arslan et al. has reported that supraglottic airway device was inserted after the induction of general anesthesia with 1 μg/kg fentanyl, 3 mg/kg propofol and 0.6 mg/kg rocuronium, and then facilitate tracheal intubation in morbidly obese patients [12]. Shiraishi reported an observational study to show a safer method of airway management in obese patients by performing awake insertion (intravenous injection of 20-40 μg/kg midazolam and 1-2 μg/kg fentanyl) of the supraglottic airway device allowing spontaneous ventilation to facilitate subsequent tracheal intubation [13]. However, the optimum anesthetics for supraglottic airway device insertion with spontaneous breathing in obese patients have not been standardized.

Although intravenous anesthesia is common, inhalational induction is a valid alternative, and for obese patients sevoflurane alone can provide all the components of general anesthesia induction with fast onset and offset. Sevoflurane as a non-pungency inhaled anesthetic with minimal respiratory irritant characteristics is suitable for anesthesia induction and supraglottic airway device insertion. Meanwhile, sevoflurane provides better hemodynamic stability and lower incidence of apnea as compared with intravenous agents, such as propofol. The ED_50_ and ED_95_ of sevoflurane required for supraglottic airway device insertion in lean patients and children are now known [14–16]. However, no study has explored optimum end-tidal concentration of sevoflurane for supraglottic airway device insertion allowing spontaneous breathing in 50% of obese patients. We considered that the anesthesia induction with sevoflurane for supraglottic airway device insertion which may allow spontaneous breathing to keep with continuous oxygenation for obese patients. Therefore, we designed this study to find out the minimum alveolar concentration of end-tidal sevoflurane required for the supraglottic airway device Blockbuster™ insertion in obese patients.

## Methods

The present study was approved by the Institutional Ethics Committee of Beijing Friendship Hospital, China (Ethics Committee number: 2016-P2–059-01), and registered with Chinese Clinical Trial Registry (http://www.chictr.org.cn; registration NO.: ChiCTR-IPR-16009071). With written informed consent, 30 obese patients with body mass index (BMI) 30–50 kg/m^2^, American Society of Anesthesiologists class I-II, aged 18–60 years old, scheduled for bariatric surgery in our central surgery unit were enrolled. Patients were not recruited if they were associated with asthma, symptoms of upper respiratory infection, neck radiation change, neck mass, unstable cervical spine, limited or severely limited jaw protrusion, interincisor distance less than 3 cm, severe obstructive sleep apnea syndrome (with an apnea-hypopnea index greater than 30 episodes per hour) [17], history of gastroesophageal reflux, or if they were allergy or sensitivity to volatile anesthetics.

All participants were fasted at least 8 h. Standard anesthesia monitoring, including electrocardiogram, non-invasive blood pressure, pulse oxygen saturation (SpO_2_) and bispectral index (BIS), was applied after the patients arriving in the operating room. The respiratory rate, tidal volume, end-tidal carbon dioxide and the concentrations of inhaled and exhaled sevoflurane were supervised. After an intravenous catheter insertion, the lactate ringer’s solution was started to infuse. The patients were given 1 mg penehyclidine hydrochloride intravenously.

Patients were positioned in the recommended ramp position (with the tragus of the ear level with the sternum, and the arms away from the chest) [18]. All patients were preoxygenated with 100% oxygen until the end-tidal oxygen fraction is 0.87. After that the circuit was filled with 5% sevoflurane at a fresh oxygen flow of 6 l per min for 1 min. And then anesthesia was induced with 5% sevoflurane via facemask with a fresh oxygen flow of 6 L/min. The inhaled concentration of sevoflurane was altered to obtain the target end-tidal concentration (2.5% in the first obese patient) after eyelash reflex loss. The oropharyngeal airway will be used in case of upper airway obstruction during the period of induction. The ratio of the measured end-tidal to target end-tidal concentration was maintained at 0.9 to 1.0 for at least 5 min to insure the equilibration between the alveolar gas tensions, arterial blood and cerebral tissue. And then, the insertion of supraglottic airway device Blockbuster™ (Tuo Ren Medical Instrument Co., Ltd., Changyuan City, China) was attempted without using neuromuscular blocking drugs [7]. A size 4 of supraglottic airway device was selected for all patients. The sevoflurane vaporizer was turned off and the fresh gas flow was stopped simultaneously during supraglottic airway device insertion.

Target sevoflurane concentration was set by one study assistant. All deflated supraglottic airway device Blockbuster™ were inserted by an experienced anesthesiologist, who had performed over 100 times. The responses of patients were evaluated by the same anesthesiologist who was blinded to the concentrations when the supraglottic airway device was inserted. The response by the patient to supraglottic airway device Blockbuster™ insertion was classified as either ‘movement’ or ‘no-movement’. Insertion of the supraglottic airway device Blockbuster™ appeared with bucking, coughing, laryngospasm or gross purposeful limb movement within 1 min of insertion was labeled as ‘movement’. Otherwise, in the paucity of any of the aforementioned problems, supraglottic airway device Blockbuster™ insertion was labeled as ‘no-movement’. Jaw relaxation was also evaluated according to Muzi score to assess the condition of supraglottic airway device Blockbuster™ insertion (1: fully relaxed, 2: mild resistance, 3: tight but could be opened, 4: closed requiring a dose of propofol) [15]. A Muzi score of 3 or 4 were also included in ‘movement’ response, otherwise, defined as ‘no-movement’ response. The additional 1 mg/kg propofol according to lean body weight will be administered immediately, if any body movements happened immediately before or after supraglottic airway device Blockbuster™ insertion. The insertion conditions were only determined for the first attempts. The incidence of apnea and apnea time (from the last spontaneous breath after sevoflurane administration to the first spontaneous breath) were recorded according to the end-tidal capnography. The apnea was defined as absence of chest movement and end-tidal capnography. If the time of apnea is over 60 s, assisted-control ventilation was adopted to avoid the oxygen desaturation. In addition, respiratory rates, tidal volume, SpO_2_, BIS variables, and baseline of respiration were also recorded. Patients were asked whether they had any recall of events or not after the surgery.

After the supraglottic airway device Blockbuster™ was connected to the breathing circuit, it allows patient to breathe spontaneously, and then the volatile anesthetic of sevoflurane was stopped to inhale. At this time, fibreoptic bronchoscope was used to observe the glottis counterpoint and to confirm correct supraglottic airway device placement. The position of the supraglottic airway device Blockbuster™ was determined by a fibreoptic scope at a position 1 cm proximal to the end of the airway tube. The fibreoptic position of the airway tube was recorded as a score using an established scoring system (grade I: vocal cords not seen, grade II: vocal cords plus anterior epiglottis seen, grade III: vocal cords plus posterior epiglottis seen, and grade IV: only vocal cords seen) [19]. After the glottis counterpoint were visualised, general anesthesia (2 mg/kg propofol, 0.3–0.4 μg/kg sufentanil, and 0.6 mg/kg rocuronium according to ideal body weight) was induced following a secure airway was confirmed. And then, oropharyngeal leak pressure was measured as follows: fresh oxygen flow was maintained at 6 L/min and the expiratory valve of the circle system was closed; the pressure was rising to an equilibrium (maximum pressure was limited to 40 cmH_2_O); the pressure was recorded at which the dial reached equilibrium [20]. A 14-French size well-lubricated gastric tube was inserted via the drainage tube; the correct placement of gastric tube was assessed by suction of fluid; the success of insertion was recorded. And then, the fibreoptic-guided tracheal intubation was performed with the suppraglottic airway device Blockbuster™ as a conduit.

In this study, the modified Dixon’s up-and-down method was adopted to determine the minimum alveolar concentration of sevoflurane for the supraglottic airway device Blockbuster™ insertion in obese patients, which has been applied previously in similar for probing anesthetic techniques of the airway device insertion according to the patients’ response [21–23]. Dixon’s up-and-down design of experiments yielded an estimate of the median threshold for all-or-nothing responses, which is sought but cannot be measured directly [24]. The target end-tidal sevoflurane concentration was started at 2.5% and it was altered by 0.5% depending on the previous patient’s response to supraglottic airway device Blockbuster™ insertion [14, 15]. If the supraglottic airway device Blockbuster™ insertion appeared with ‘movement’ response, the target end-tidal sevoflurane concentration for supraglottic airway device Blockbuster™ insertion was increased by 0.5% for the next patient. Otherwise, the target end-tidal sevoflurane concentration was decreased by 0.5% for the next patient.

The sample size of this study was based on the fact that a minimum of eight independent pairs of participants showing a crossover point from a ‘movement’ response to a ‘no-movement’ response required for the statistical analysis [24, 25]. In similar studies in the field of airway device insertion, the number of crossover points varies from six to eight, with six crossover points being most common [23, 26, 27]. For this study, eight crossovers were considered sufficient to determine the ED_50_ of sevoflurane required to insert the suppraglottic airway device Blockbuster™.

The ED_50_ of sevoflurane was determined by calculating the average of the crossover midpoints from ‘movement’ to ‘no-movement’ response obtained in all participants using the Dixon’s up-and-down method. The standard deviation (SD) of the ED_50_ represented the SD of the crossover midpoints. We further analysed our data using probit regression curves to determine the probability of “successful insertion” relative to end-tidal sevoflurane concentration and to obtain end-tidal sevoflurane concentrations where 50% (ED_50_) and 95% (ED_95_) with 95% confidence interval (CI) of the suppraglottic airway device Blockbuster™ insertion attempts were successful. Respiratory index were analysed with repeated measures of analysis of variance (ANOVA). Patients’ characteristics are represented as mean (SD), median (IQR [range]) or number (proportion). *P* value less than 0.05 was considered significant. These data were analysed using SPSS version 17.0 (SPSS Inc., Chicago, IL, USA).

## Results

**Fig. 1:**
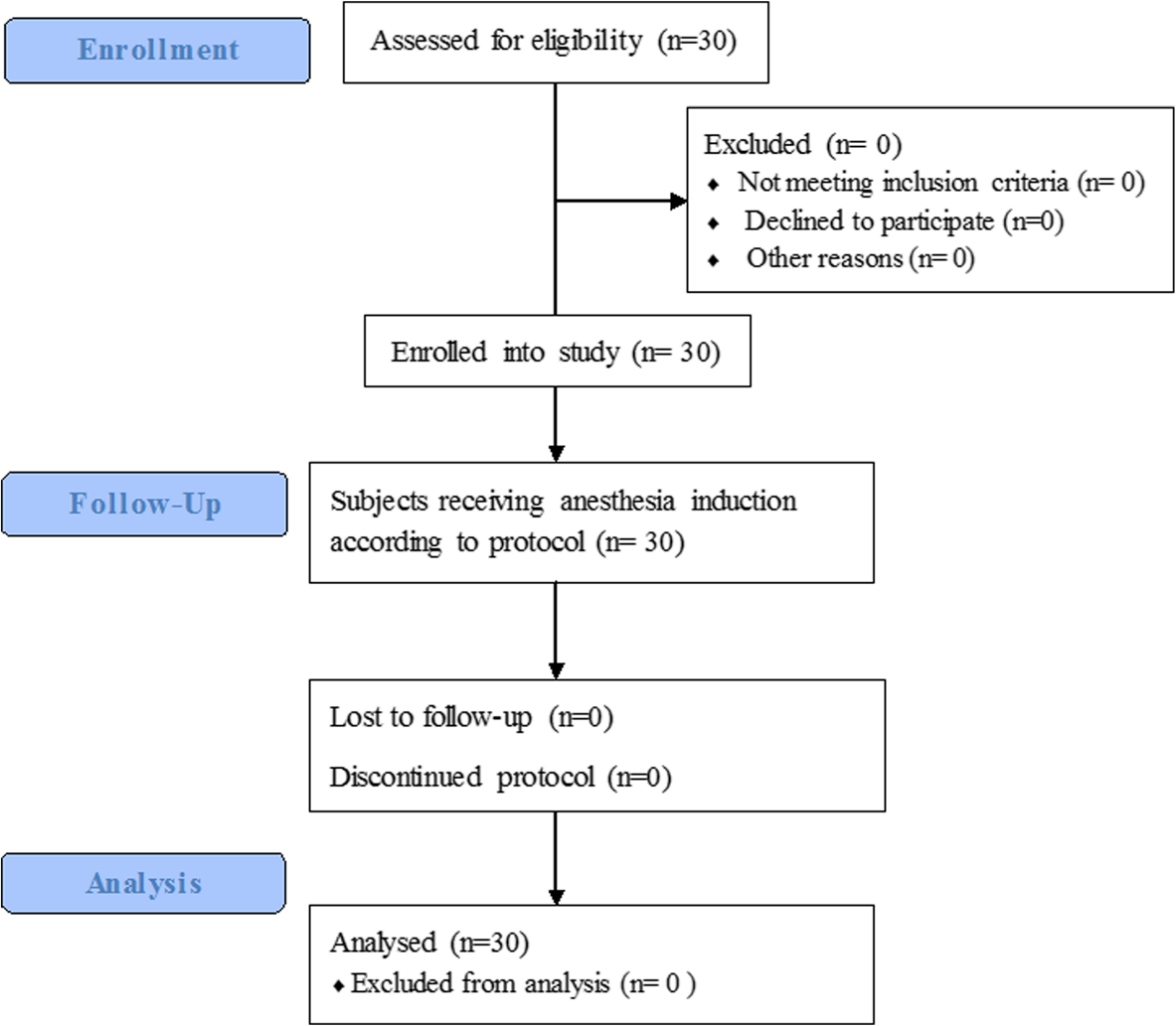
Flow diagram of obese patients recruitment

Thirty obese patients were enrolled in this study, and all of them completed the study (Fig. 1). Patients’ characteristics and airway features are presented in Table 1.

**Table 1:** Baseline characteristics of obese patients

	Obese patients (*n* = 30)
Age; years	31.8 (7.47)
Gender; female	22 (73.3%)
Height; cm	166 (6.27)
Weight; kg	105 (17.4)
BMI; kg/m^2^	37.9 (5.17)
Neck circumference; cm	41.4 (3.52)
Thyromental distance; cm	8.97 (0.947)
Interincisor distance; cm	5.77 (0.640)
Mallampati class	
I	3 (10.0%)
II	14 (46.7%)
III	12 (40.0%)
IV	1 (3.33%)
Obstructive sleep apnea syndrome	19 (63.3%)
mild	13 (43.3%)
moderate	6 (20%)
Apnea-hypopnea index	10.29 (9.86)
Hypertension	8 (26.7%)
Diabetes mellitus	8 (26.7%)
Hyperlipemia	8 (26.7%)

Values are expressed as mean (SD) or numbers (proportion)

*BMI* body mass index

**Fig. 2:**
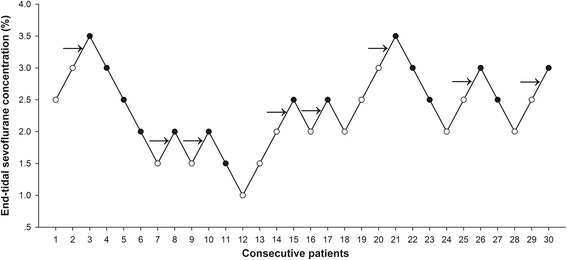
Responses of obese patients with a modified Dixon’s up-and-down method. Responses of 30 consecutive obese patients to supraglottic airway device Blockbuster™ insertion and the end-tidal concentrations of sevoflurane in oxygen with a modified Dixon’s up-and-down method. Arrows indicate the mid-point dose of all independent pairs of patients who manifested cross-over from ‘movement’ (○) to ‘no-movement’ (●) response

**Fig. 3:**
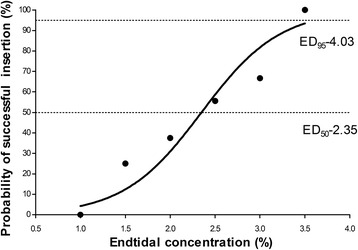
Dose-response curves of sevoflurane for supraglottic airway device Blockbuster™ insertion in obese patients. The curves plotted from probit regression analysis of individual end-tidal sevoflurane concentrations and the reactions to supraglottic airway device Blockbuster™ insertion in obese patients. The ED_50_ and ED_95_ were 2.35% (95% CI 1.28–3.42%) and 4.03% (95% CI 3.16–17.83%), respectively

Dose-response data obtained by Dixon’s up-and-down method was illustrated in Fig. 2. The minimum alveolar concentration of sevoflurane for supraglottic airway device Blockbuster™ insertion in obese patients calculated by the up-and-down method was 2.50 ± 0.60%. The probit regression of the dose-response curve for each patient indicating the probability of successful insertion vs. end-tidal concentration of sevoflurane is shown in Fig. 3. The predicted ED_50_ and ED_95_ of sevoflurane for supraglottic airway device Blockbuster™ insertion were 2.35 (95% CI 1.28–3.42) % and 4.03 (95% CI 3.16–17.83) %, respectively.

The characteristics of obese patients experienced anesthesia induction and supraglottic airway device Blockbuster™ insertion are also summarized in Table 2. Four patients developed apnea and two of them experienced apnea after receiving propofol. The incidence of apnea was 13.3%, and the longest time of apnea was 47.9 s. The lowest SpO_2_ during the procedure was 94% in one patient. No patient experienced oxygen desaturation (defined as SpO_2_ less than 92%) during this study. The respiratory rates and tidal volume were significantly different at different measure point (*P* < 0.001).

**Table 2:** Characteristics of anesthesia induction and postoperative interview in obese patients

	Obese patients (*n* = 30)
Anesthesia induction period	
Laryngospasm	0
Coughing	4 (13.3%)
Purposeful limb movement	9 (30.3%)
Muzi score > 2	15 (50.0%)
Number of patients with apnea	4 (13.3%)
Apnea time (s)	38.2 (7.37)
Longest apnea time (s)	47.9
Lowest SpO_2_ during tracheal intubation (%)	97.5 (97–98.3 [94–99])
Successful insertion at first attempt	15 (50%)
Successful insertion at second attempt	13 (43.3%)
Successful insertion at third attempt	2 (6.67%)
Respiratory rate*	
Before induction	14 (12–15 [10–22])
1 min after induction	14 (11–15 [9–16])
1 min before supraglottic airway device insertion	16 (14–18 [10–21])
immediately before supraglottic airway device insertion	17 (16–18 [12–22])
1 min after supraglottic airway device insertion	16 (14–18 [10–22])
Tidal volumes^†^	
Before induction	605 (101)
1 min after induction	412 (93.6)
1 min before supraglottic airway device insertion	361 (90.1)
immediately before supraglottic airway device insertion	339 (79.2)
1 min after supraglottic airway device insertion	366 (82.3)
Postoperative interview	
Pleasant induction	30
Repeat same anesthetic technique	30

Values are expressed as mean (SD), numbers (proportion), or median (IQR [range])

*P < 0.001; ^†^ *P* < 0.001

The hemodynamic data and Bispectral index are shown in Table 3. The BIS values were similar immediately before supraglottic airway device Blockbuster™ insertion in the ‘movement’ (53 ± 10) and ‘no-movement’ patients (51 ± 8) (*P* = 0.295). According to the postoperative visit, no patients recalled of events during anesthesia induction and supraglottic airway device Blockbuster™ insertion in this study. All patients were pleasant with our induction techniques, and anesthesiologists felt comfortable. Data regarding oropharyngeal leak pressure, fibreoptic position of the airway tube and success of insertion of gastric tube were presented in Table 4. There were no clinically significant events of laryngospasm, aspiration, hypertension, hypotension, tachycardia or bradycardia related to anesthesia induction and supraglottic airway device Blockbuster™ insertion requiring treatment. No patient reported dysphagia or dysphonia. The incidence of complication related to sevoflurane induction and supraglottic airway device Blockbuster™ insertion are provided in Table 5.

**Table 3:** Changes in haemodynamic data and Bispectral index before and after insertion of a supraglottic airway device following induction of anesthesia with sevoflurane

Variable		Obese patients (*n* = 30)	*P* value
Systolic BP (mmHg)	Before induction	139 (11)	< 0.001
	1 min after induction	134 (12)	
	before supraglottic airway device insertion	130 (14)	
	after supraglottic airway device insertion	125 (13)	
Diastolic BP (mmHg)	Before induction	81 (9)	< 0.001
	1 min after induction	75 (8)	
	before supraglottic airway device insertion	71 (9)	
	after supraglottic airway device insertion	69 (8)	
Heart rate (bpm)	Before induction	71 (12)	0.004
	1 min after induction	69 (11)	
	before supraglottic airway device insertion	66 (11)	
	after supraglottic airway device insertion	71 (13)	
BIS	Before induction	93 (4)	< 0.001
	1 min after induction	57 (8)	
	before supraglottic airway device insertion	52 (9)	
	after supraglottic airway device insertion	58 (11)	

Values are expressed as mean (SD)

*BIS* Bispectral index

**Table 4:** Fibreoptic position of the airway tube, oropharyngeal leak pressure and the success of the insertion of gastric tube

	Obese patients (*n* = 30)
Fibreoptic position	
I	3 (10.0%)
II	5 (16.7%)
III	9 (30.0%)
IV	13 (43.3%)
Oropharyngeal leak pressure (cm H_2_O)	30.4(3.10)
Gastric tube insertion success	30 (100%)

Values are expressed as mean (SD) or numbers (proportion)

**Table 5:** Incidence of complications related to anesthesia induction and supraglottic airway device insertion

	Obese patients (*n* = 30)
Complications during induction	
Excitatory movements	3 (10.0%)
Laryngospasm	0
Aspiration	0
Hiccup	0
Complications during supraglottic airway device insertion	
Coughing	4 (13.3%)
Laryngospasm	0
Aspiration	0
Nerves injury	0

Values are expressed as mean (SD) or numbers (proportion)

## Discussion

To our knowledge, this study is the first attempt to determine the ED_50_ of end-tidal sevoflurane concentration required for supraglottic airway device Blockbuster™ insertion with spontaneous breathing in obese patients. The result showed that anesthesia induction with sevoflurane can provided acceptable conditions for supraglottic airway device Blockbuster™ insertion allowing spontaneous breathing in obese patients.

Obese patients are at higher risk of difficult mask ventilation as well as difficult tracheal intubation [28, 29]. Oxygenation is the first priority in the difficult airway management [30]. In addition, oxygen desaturation is rapidly following the cessation of breathing. Therefore, the way to oxygenate continually for obese patient is conclusive. Supraglottic airway devices, as viable tools for airway management, have been recommended to provide oxygenation in the way of both routine use and rescue airway management [4, 31]. Previous studies have reported the anesthetic techniques for supraglottic airway device insertion in children and lean patients [16, 32, 33]. However, the dose of anesthetic agents required to insert supraglottic airway devices is much less fully investigated in obese patients. Zaballos et al. showed that sevoflurane alone can provide satisfied condition for laryngeal mask airway Supreme (Laryngeal Mask Company Limited, Singapore, Singapore) insertion, and the end-tidal sevoflurane concentration for laryngeal mask airway Supreme insertion in 50% of lean patients is 3.03% [15]. Kodaka et al. found that the end-tidal sevoflurane concentrations were 2.36 and 2.82% for insertion of the laryngeal mask airway Classic (Laryngeal Mask Company, Henley-on-Thames, UK) and laryngeal mask airway ProSeal (Laryngeal Mask Company, Henley-on-Thames, UK) [14]. However, the optimum sevoflurane concentration for supraglottic airway device Blockbuster™ insertion allowing spontaneous breathing in obese patient has not been investigated. In the current study, the result showed that the minimum alveolar anesthetic concentration of sevoflurane for supraglottic airway device Blockbuster™ insertion in obese patients was 2.5%. The current study was different from previous studies in several aspects. Firstly, it was the employed patients which all of them were adult obese patients with mean age of 31.8 years in this study. However, in the study from Zaballos et al. and Kodaka et al., mean age of the patients was 50 years and 42 years. Secondly, supraglottic airway device Blockbuster™ insertion was attempted without using neuromuscular blocking drugs which was considering of keeping with spontaneous breathing during the procedure of supraglottic airway device insertion; Thirdly, airway devices adopted in this study were supraglottic airway device Blockbuster™ (Tuo Ren Medical Instrument Co., Ltd., Changyuan City, China) instead of the laryngeal mask airway Classic or laryngeal mask airway ProSeal (Laryngeal Mask Company, Henley-on-Thames, UK). These differences could contribute to the different value of the ED_50_ of sevoflurane for supraglottic airway device insertion observed between the current study and the previous studies. However, the procedure of supraglottic airway device insertion with spontaneous breathing for obese patients was safer and the result of this study would suggest that a predicted minimum alveolar concentration of 2.5% sevoflurane for supraglottic airway device Blockbuster™ insertion allowing spontaneous breathing could be a better choice for obese patients’ airway management pending further clinical investigations.

The results of the present study showed that sevoflurane can provided acceptable conditions for supraglottic airway device insertion allowing spontaneous ventilation with minimal adverse effects. The incidence of apnea was 13.3%. However, it did not cause oxygen desaturation obviously in all patients. Previous study has showed that apnea occurred in 16% of patients experienced anesthesia induction with sevoflurane for laryngeal mask insertion [34]. Pancaro et al. have reported that the incidence of apnea during anesthesia induction of sevoflurane ranged from 20% to 68% [35]. For one thing, the additional propofol was adopted in ‘movement’ patients, which may have resulted in the increased incidence of apnea. For another thing, this may be because of the induction of anesthesia with inhaled sevoflurane concentration. Anesthesia was induced with inhaled sevoflurane up to 5% in this study, and the inhaled concentration of sevoflurane was decreased to obtain the target end-tidal concentration after eyelash reflex loss. Apnea during anesthesia induction with sevoflurane is correlated with its mode of administration. The faster and higher concentration of sevoflurane is inhaled the higher is the probability the patient will suffer with apnea [35].

During this study, the mean oropharyngeal leak pressure in this study was 30.4 cm H_2_O, and it is similar to previous study with a mean oropharyngeal leak pressure at 28cmH_2_O for laryngeal mask airway Supreme [36]. High oropharyngeal leak pressures indicate well airway protection and success supraglottic airway device placement [20]. And also, there were no clinically significant events of airway complications happened during this study.

The airway-management with a supraglottic airway device is also difficult in obese patients, especially as an airway rescue had an increased failure rate [18]. Therefore, it is necessary to explore a method to insert a supraglottic airway device with spontaneous breathing for obese patients. Inhalational induction is an accepted technique with spontaneous breathing for the difficult airway management [37, 38]. This study, intended to present practical values for obese patients, could help clinicians who prepared to use a supraglottic airway device in such a scenario. The method of anesthesia induction made patients feel relax and avoided the distress caused by the performance of supraglottic airway device insertion. The passive situation as ‘can’t intubate, can’t ventilate’ could be avoided by using this induction technique which makes anesthetists feeling comfortable and confident in the airway management of obese patients.

There were several limitations in the present study. Firstly, we limited our study to obese patients (BMI 30–50 kg/m^2^) scheduled for bariatric surgery. It is uncertain whether our results could be extended to morbidly obese patients with higher BMI (> 50 kg/m^2^). Secondly, this study was limited to Asian and the ED_50_ is likely to be affected in different racial groups. Thirdly, the age of enrolled patients raged from 18 to 56, and the ED_50_ may be different for obese children and older patients. In addition, we used an increment of 0.5% sevoflurane in our study, even if appropriate increments should be 10 or 15% of the starting dose or the expected minimum alveolar concentration (MAC) value according to Paul and Fisher [39]. However, the increment of 0.5% is often used in similar studies, and this step permitted us to compare the results with that of other studies. Finally, sevoflurane ED_50_ for supraglottic airway device insertion cannot apply to clinical practice directly. Clinicians would be much more interested in ED_95_ on account of its greater clinical significance. The ED_95_ of sevoflurane was obtained by using probit regression analysis which has been applied in literature using Dixon’s up-and-down method [40–42]. However, the method shows the possibility of placing a supraglottic airway device Blockbuster™ without movement response ignoring other parameters (respiratory parameters, BIS and hemodynamic values). Therefore, further studies should be warranted to investigate the ED_95_ by a larger number and detailed comparison with wider range of sevoflurane concentrations.

## Conclusion

We found that the supraglottic airway device Blockbuster™ can be successfully inserted allowing spontaneous breathing in 50% of the obese patients at a predicted end-tidal sevoflurane concentrations of 2.5% with minimal adverse effects.

## Abbreviations

BIS: Bispectral index
BMI: Body mass index
CI: Confidence interval
ED_50_: Effect-site concentration in 50%
ED_95_: Effect-site concentration in 95%
SpO_2_: Pulse oxygen saturation
